# Intergenerational concordance of brain structure between depressed mothers and their never-depressed daughters

**DOI:** 10.1192/j.eurpsy.2023.511

**Published:** 2023-07-19

**Authors:** F. Minami, J. Hirano, R. Ueda, A. Takamiya, M. Yamagishi, K. Kamiya, M. Mimura, B. Yamagata

**Affiliations:** ^1^Neuropsychiatry, Keio University School of Medicine; ^2^ Warakukai Medical Corporation; ^3^Radiation Technology, Keio University School of Medicine, Tokyo, Japan; ^4^Department of Neurosciences, Neuropsychiatry, KU Leuven, Leuven Brain Institute, Leuven, Belgium; ^5^Ebis Medical Clinic, Tokyo, Japan

## Abstract

**Introduction:**

Parents have significant genetic and environmental influences, which are known as intergenerational effects, on the cognition, behavior, and brain of their offspring. These intergenerational effects are observed in patients with mood disorders, with a particularly strong association of depression between mothers and daughters.

**Objectives:**

The main purpose of our study was to investigate female-specific intergenerational transmission patterns in the human brain among patients with depression and their never-depressed offspring.

**Methods:**

We recruited 78 participants from 34 families, which included remitted parents with a history of depression and their never-depressed biological offspring. We used source-based and surface-based morphometry analyses of magnetic resonance imaging data to examine the degree of associations in brain structure between four types of parent-offspring dyads (i.e. mother-daughter, mother-son, father-daughter, and father-son).

**Results:**

Using independent component analysis, we found a significant positive correlation of gray matter structure between exclusively the mother-daughter dyads within brain regions located in the default mode and central executive networks, such as the bilateral anterior cingulate cortex, posterior cingulate cortex, precuneus, middle frontal gyrus, middle temporal gyrus, superior parietal lobule, and left angular gyrus. These similar observations were not identified in other three parent-offspring dyads.

**Conclusions:**

The current study provides biological evidence for greater vulnerability of daughters, but not sons, in developing depression whose mothers have a history of depression. Our findings extend our knowledge on the pathophysiology of major psychiatric conditions that show sex biases and may contribute to the development of novel interventions targeting high-risk individuals.

**Disclosure of Interest:**

None Declared

